# Neonatal Cholestasis: A Rare and Unusual Presentation of Pituitary Stalk Interruption Syndrome

**DOI:** 10.1155/2021/6161508

**Published:** 2021-05-22

**Authors:** R. El Qadiry, A. Ouayad, H. Nassih, A. Bourrahouat, I. Ait Sab

**Affiliations:** Pediatric B Department, Mother-Child Pole, Mohammed VI University Hospital, Marrakesh, Morocco

## Abstract

Pituitary stalk interruption syndrome (PSIS) is a very rare entity, and the clinical manifestations are nonspecific. Neonatal cholestasis due to endocrine disorders is rare and poorly recognized. Our case report describes a case of PSIS in a Moroccan infant revealed by isolated neonatal cholestasis, which is an unusual presentation in children. *Case report*. A 40-day-old girl was admitted to our department for progressive cholestatic jaundice appeared on the third day of life. She was born from a non-consanguineous marriage, and her prenatal and perinatal history went without incident. Physical examination showed icteric skin and sclera, without hepatomegaly. Analysis of pituitary hormones revealed panhypopituitarism. On brain magnetic resonance imaging (MRI), the pituitary stalk was absent, the posterior pituitary was ectopic, and the anterior pituitary was hypoplastic. The patient was diagnosed with interrupted pituitary stalk syndrome. The treatment consisted of hormone replacement with rapid improvement of her clinical condition. *Conclusion*. Panhypopituitarism, a consequence of PSIS, is a rare cause of neonatal cholestasis. However, pediatricians should keep this syndrome in mind for patients who present with neonatal cholestasis.

## 1. Introduction

Pituitary stalk interruption syndrome (PSIS) is a rare congenital anomaly, characterized by pituitary hormone deficiencies along with radiological features of a thin or interrupted pituitary stalk, an ectopic or absent posterior pituitary, or a hypoplastic or absent anterior pituitary [1].

Clinical signs are variable and nonspecific, which may lead to delayed diagnosis. Neonatal cholestasis due to endocrine disorders is rare and poorly recognized.

Our case report describes a case of PSIS in a Moroccan infant revealed by isolated neonatal cholestasis, which is an unusual presentation in children.

## 2. Case

A 40-day-old girl was admitted to our department for progressive cholestatic jaundice appeared on the third day of life. The child was born at full term from a non-consanguineous marriage with a birth weight of 3500 g (25–50th birth weight percentile). Her perinatal history went without incident; no episodes of hypoglycemia or seizures were noted. Family history did not involve hepatobiliary disease or endocrine disorder.

Physical examination showed icteric skin and sclera, with dark urine and pale stool, without hepatomegaly or splenomegaly. No obvious facial dysmorphism was observed.

Laboratory results revealed total bilirubin of 154 mg/l with 136 mg/l corresponding to conjugated bilirubin. The serum alkaline phosphatase (ALP) was elevated (719 IU/L) (N: 0–449), while gamma-glutamyl transpeptidase (*γ*-GTP), serum liver enzymes, and prothrombin time (PT) were within the normal range (*γ*-GTP: 38 UI/L, AST: 58 UI/L, ALT: 22 IU/L, and PT: 84%). Plasma glucose level was normal (85 mg/dL).

Abdominal ultrasound and magnetic resonance cholangiopancreatography showed normal liver and gallbladder anatomy and patent hepatobiliary tract, ruling out the possibility of biliary atresia. Amino acid analyses did not show any abnormalities. Test screening for hepatitis viruses (TORCH infections) was negative. Hemoglobin was 9.6 g/dl, white blood cells were 12400/mm^3^, CRP was 18 mg/l (<6), and urine cytobacteriological examination showed a urinary tract infection with *Klebsiella pneumoniae*, and she had been treated successfully, but jaundice persisted.

**Figure 1:**
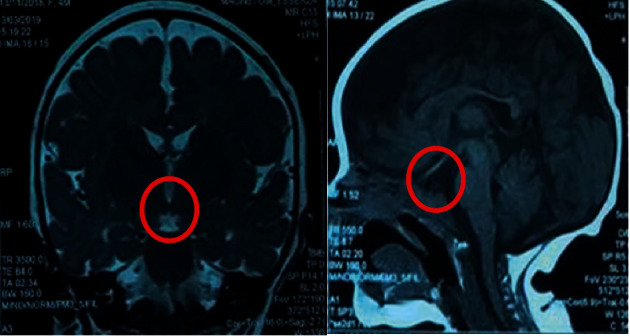
MRI of the head revealed a small anterior pituitary gland, an invisible stalk, and an ectopic posterior lobe.

Then, pituitary hormonal profiles showed a low morning serum cortisol level at 1.30 *μ*g/dl (reference range: 8, 7–22, 40 *μ*g/dl) and a normal plasma adrenocorticotropic hormone (ACTH) level at 8.9 ng/l (reference range: 5–60 ng/l), indicating secondary adrenal insufficiency. Thyroid function tests showed a low free T4 level of 8.6 pmol/L (reference range: 12–22 pmol/L) and a normal TSH level of 3.61 mIU/L, suggesting central hypothyroidism. A low, insulin-like growth factor-1 (IGF-1) level <10 ng/ml (reference range: 19–104 ng/l) in combination with deficiency of more than two pituitary hormones was highly indicative of growth hormone deficiency. A magnetic resonance imaging (MRI) of the head revealed a small anterior pituitary gland, an invisible stalk, and an ectopic posterior lobe (Figure 1). The infant was diagnosed with pituitary stalk interruption syndrome.

The treatment consisted of hormone replacement with hydrocortisone (15 mg/m^2^/day) and subsequently L-thyroxin (3.5 *μ*g/kg/day). GH therapy is not yet initiated.

**Figure 2:**
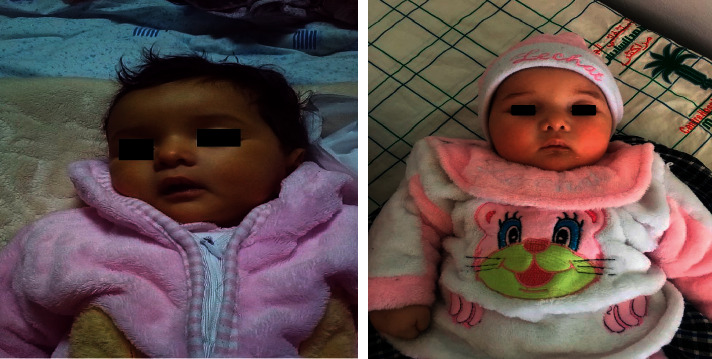
After 3 months of follow-up, the jaundice disappeared completely.

After 3 months of follow-up, the jaundice disappeared completely and the growth was still within the norms (Figure 2).

## 3. Discussion

Pituitary stalk interruption syndrome (PSIS) is a rare entity, with incidence rate of 0.5/1,000,000 per birth. The exact mechanism of this pathology is unclear. It can occur in a variety of ways. Earlier signs may include hypoglycemia, jaundice, cryptorchidism, and seizures [2].

Panhypopituitarism, a consequence of PSIS, is a rare cause of cholestasis in infants. In addition, it is generally neglected and misdiagnosed in infants with isolated cholestasis [3]. Our case underlines the importance of endocrine disorders as one of the etiologies of neonatal cholestasis. However, in the absence of physical findings suggestive of hypopituitarism, the diagnosis is less apparent.

While hormone deficiency is probably the strongest explanation for neonatal cholestasis in PSIS patients, it is not exactly known how hormone deficiencies cause cholestasis. It has been suggested that thyroid hormone and cortisol can influence the bile acid-independent bile flow and bile formation. In addition, growth hormone can modulate the biosynthesis and secretion of bile acids, and it has been shown that bleary flow recovers after hydrocortisone and GH treatment. However, cholestasis in isolated GH deficiency was never reported. For our patient, the cholestasis resolved before GH therapy instauration [3, 4].

Diagnosis can be achieved after clinical suspicion of hypopituitarism symptoms; laboratory tests and exact diagnosis can be achieved via imaging methods. MRI, with detailed hormone analysis, is the key diagnostic method for the exact diagnosis [5].

After diagnosis, hormonal therapy should be started first with cortisol and then thyroxine. This is because restoration of a euthyroid state can destabilize a patient with ACTH deficiency. GH therapy is often required later in infancy [6].

## 4. Conclusion

Pituitary stalk interruption syndrome is a very rare entity, and the clinical manifestations are nonspecific. Cholestasis may be the only clinical feature in the neonatal period. Consequently, many cases are exposed to extensive diagnostic tests before coming up to the suspicion of an endocrine disorder, and the treatment is delayed exposing patients to serious complications. Therefore, pediatricians should keep this syndrome in mind for patients who present with neonatal cholestasis.
